# High Sensitivity TSS Prediction: Estimates of Locations Where TSS Cannot Occur

**DOI:** 10.1371/journal.pone.0013934

**Published:** 2010-11-15

**Authors:** Ulf Schaefer, Rimantas Kodzius, Chikatoshi Kai, Jun Kawai, Piero Carninci, Yoshihide Hayashizaki, Vladimir B. Bajic

**Affiliations:** 1 Computational Bioscience Research Center (CBRC), King Abdullah University of Science and Technology, Thuwal, Kingdom of Saudi Arabia; 2 Division of Physical Sciences and Engineering, King Abdullah University of Science and Technology, Thuwal, Kingdom of Saudi Arabia; 3 Genome Exploration Research Group (Genome Network Project Core Group), RIKEN Genomic Sciences Center (GSC), RIKEN Yokohama Institute, Yokohama, Kanagawa, Japan; 4 Genome Science Laboratory, Discovery Research Institute, RIKEN Wako Institute, Wako, Saitama, Japan; King Abdullah University of Science and Technology, Saudi Arabia

## Abstract

**Background:**

Although transcription in mammalian genomes can initiate from various genomic positions (e.g., 3′UTR, coding exons, etc.), most locations on genomes are not prone to transcription initiation. It is of practical and theoretical interest to be able to estimate such collections of non-TSS locations (NTLs). The identification of large portions of NTLs can contribute to better focusing the search for TSS locations and thus contribute to promoter and gene finding. It can help in the assessment of 5′ completeness of expressed sequences, contribute to more successful experimental designs, as well as more accurate gene annotation.

**Methodology:**

Using comprehensive collections of Cap Analysis of Gene Expression (CAGE) and other transcript data from mouse and human genomes, we developed a methodology that allows us, by performing computational TSS prediction with very high sensitivity, to annotate, with a high accuracy in a strand specific manner, locations of mammalian genomes that are highly unlikely to harbor transcription start sites (TSSs). The properties of the immediate genomic neighborhood of 98,682 accurately determined mouse and 113,814 human TSSs are used to determine features that distinguish genomic transcription initiation locations from those that are not likely to initiate transcription. In our algorithm we utilize various constraining properties of features identified in the upstream and downstream regions around TSSs, as well as statistical analyses of these surrounding regions.

**Conclusions:**

Our analysis of human chromosomes 4, 21 and 22 estimates ∼46%, ∼41% and ∼27% of these chromosomes, respectively, as being NTLs. This suggests that on average more than 40% of the human genome can be expected to be highly unlikely to initiate transcription. Our method represents the first one that utilizes high-sensitivity TSS prediction to identify, with high accuracy, large portions of mammalian genomes as NTLs. The server with our algorithm implemented is available at http://cbrc.kaust.edu.sa/ddm/.

## Introduction

The annotation of mammalian genomes is far from complete. Sequencing of full-length cDNA libraries, generation of thousands of ESTs, and later tag approaches like CAGE [1] and GIS [2] or SAGE [3], [4] have provided us with information on transcripts and their transcription start site (TSS) locations. Although transcription in mammalian genomes can initiate at various positions (e.g. coding exons, 3′UTR, etc.) [5], [6], it does not initiate randomly. Large segments of genomes are not prone to transcription initiation, while the remaining parts seem to make a more suitable environment for such events. A detailed analysis of the TSS neighborhood [7] shows that there are a lot of regularities in the regions immediately surrounding TSSs. It is also known that many regions in mammalian genomes are considered to be gene deserts [8], [9] (i.e. regions highly depleted of genes), while, at the same time, many other segments of the genome are rich with genes, such as for example human chromosome 22 [10], [11]. A traditional way to interpret these gene dense and gene desert regions is in the convenient terms of GC-richness of isochores on the mammalian genomes [12].

The efforts of the scientific community have provided a large amount of transcript data that has allowed the very precise determination of a large number of TSSs in mouse and human genomes [1], [2], [13], [14]. We observed, based on the analysis of the upstream and downstream properties of the TSS surroundings [7] that TSS locations in both mouse and human follow certain rules that confine these TSSs to particular genomic regions. We utilized this idea and extended it further with the aim to develop the Dragon TSS Desert Masker (DDM), an algorithm that can, with high accuracy, demarcate in a strand specific manner, positions on mammalian (mouse and human) genomes as those that can initiate transcription (transcription initiation active regions, TIARs) and those that are not likely to do so (non-TSS locations, NTLs). In practice, DDM operates as a TSS finding algorithm tuned to ∼100% sensitivity. Thus, the genomic locations which DDM does not predict as TSS represent NTLs. The remaining regions indicate TIARs that are likely to harbor the vast majority of genuine TSSs. The reliable estimation of NTLs can support a more precise RACE primer design, and can help in estimating the completeness of 5′ ends of ESTs. Consequently, NTLs can complement the annotation of promoter regions in mammalian genomes. Moreover, such information can support promoter and gene finding and help to keep more focus on regions of particular interest. For the sake of completeness, we compared DDM with several existing promoter prediction systems with regard to their ability to estimate NTLs. We show that DDM outperforms the existing TSS prediction systems in this specific task. Consequently we find DDM to be the only tool currently available to reliably identify NTL.

## Results

We define NTLs as the set of all strand-specific genomic locations that cannot initiate transcription. Regrettably, since it must be assumed that not all genuine TSS locations are known for any mammalian genome, only estimates of NTLs can be made at this point. For simplicity we will refer to these estimates as NTLs in the remainder of the text.

TIARs represent a set of genomic locations that contains the vast majority of all known TSS locations, as well as all locations that were falsely recognized as potential TSSs by DDM. In order to be able to estimate NTL we need a TSS recognition system that operates at or very near 100% sensitivity. At this level of sensitivity we can expect that the areas labeled as NTLs are indeed almost completely devoid of TSSs because all or nearly all predicted NTL locations by the system will be true non-TSS. We have developed an algorithm that is capable of achieving this.

### Algorithm

**Figure 1:**
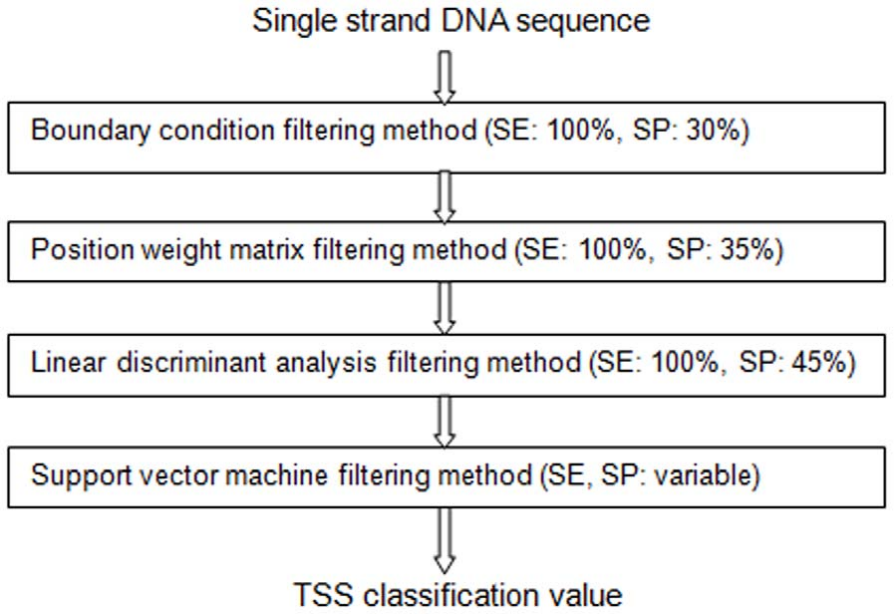
Algorithm layout. Layout of daisy-chain algorithm, performance estimates after each step in parenthesis.

Based on Fantom3 CAGE data [5] and at least one other piece of evidence for the existence of transcripts (see Methods section) we have compiled two highly accurate sets of TSS locations consisting of 98,682 TSSs for mouse and 113,814 TSSs for human. Using these sets, we analyzed compositional properties of single-stranded DNA segments covering [−100,+100] nt regions relative to the TSS. Based on these properties we designed different methods for filtering out those DNA locations that are unlikely to represent genuine TSS positions. We combined these filtering methods in a multi-staged daisy-chain algorithm that consists of four different classification phases (see Figure 1, for further details see Methods section.)

The algorithm returns a classification value for each examined DNA segment. This output classification value reflects the algorithm's prediction whether the segment contains a TSS at its centre or not. A threshold value is applied and the center (nucleotide at position +1) of the examined [−100, +100] sequence is demarcated as NTL if the classification value is below this threshold. The threshold value is usually chosen in such a way that no genuine TSS is falsely classified, but it can be modified to examine the algorithms behavior at various settings (see Methods section).

### Performance

**Figure 2:**
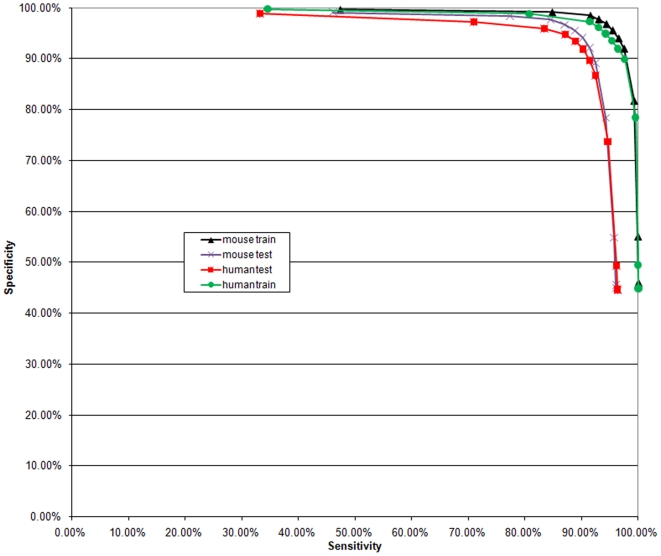
Performance curve. Sensitivity vs. Specificity trade-off curve for human and mouse average CV performance (blue and red), and performance on the whole data sets (black and green).

The algorithm was applied to mouse and human data sets. All genuine TSSs from our data were used as positive samples, while an equal amount of random DNA from the respective species served as negative samples. The performance is reported for two cases (Figure 2 and Table 1). In a 4-fold cross-validation we used a quarter of the sequences selected randomly for testing, while three quarters were used for training. Finally, we utilized the entire available data sets and the resulting models are implemented on our web-server (http://cbrc.kaust.edu.sa/ddm/).

**Table 1:** Sensitivity and Specificity values for mouse and human test and training cases.

Mouse whole set			Mouse CV			Human whole set			Human CV		
threshold	Sensitivity	Specificity	threshold	Sensitivity	Specificity	threshold	Sensitivity	Specificity	threshold	Sensitivity	Specificity
−2.50	100.00%	45.45%	−2.50	96.10%	45.01%	−2.50	100.00%	44.77%	−2.50	96.36%	44.51%
−2.00	99.99%	45.95%	−2.00	96.07%	45.55%	−2.00	100.00%	44.97%	−2.00	96.36%	44.74%
−1.50	99.91%	55.03%	−1.50	95.72%	54.82%	−1.50	99.98%	49.47%	−1.50	96.20%	49.34%
−1.00	99.22%	81.75%	−1.00	94.30%	78.40%	−1.00	99.53%	78.45%	−1.00	94.62%	73.73%
−0.50	97.47%	92.03%	−0.50	92.49%	89.16%	−0.50	97.59%	89.92%	−0.50	92.46%	86.71%
−0.25	96.50%	94.05%	−0.25	91.41%	92.10%	−0.25	96.42%	91.96%	−0.25	91.47%	89.69%
0.00	95.44%	95.63%	0.00	90.21%	94.11%	0.00	95.33%	93.58%	0.00	90.29%	91.91%
0.25	94.33%	96.86%	0.25	88.80%	95.52%	0.25	94.23%	94.94%	0.25	88.91%	93.41%
0.50	92.98%	97.81%	0.50	86.98%	96.68%	0.50	92.98%	96.17%	0.50	87.12%	94.74%
0.75	91.50%	98.51%	0.75	84.52%	97.66%	0.75	91.46%	97.29%	0.75	83.42%	95.91%
1.00	84.76%	99.16%	1.00	77.36%	98.40%	1.00	80.74%	98.83%	1.00	70.95%	97.22%
1.25	47.32%	99.65%	1.25	46.08%	99.09%	1.25	34.57%	99.76%	1.25	33.19%	98.91%

We observe that, with the models implemented on our web server, by choosing 100% sensitivity our system is able to recognize about 45% of the mouse and human random non-TSS DNA sequences correctly as those that should not initiate transcription, while with the DDM's sensitivity of 99.22% (99.53%) we are able to demarcate a remarkable proportion of 81.75% (78.45%) of the mouse (human) random non-TSS DNA sequences as those that are unlikely to initiate transcription. DDM sensitivity of 99.22% means that we are not able to recognize only 0.78% of the real TSSs from our TSS set. When DDM is recognizing 95.44% (95.33%) of TSSs from our set, at the same time it annotates 95.63% (93.58%) of the random mouse (human) sequences as unlikely to initiate transcription. When 84.76% (80.74%) of the TSSs from our set are recognized as positions likely to initiate transcription, 99.16% (98.83%) of the random non-TSS mouse (human) sequences are annotated as NTLs. For the estimates of NTLs the performance of the algorithm at very high sensitivity levels (100%) is most relevant. This is because only when the false-negative rate is equal to or very near to 0% one can meaningfully speak of the areas that were recognized as unlikely to initiate transcription to be NTLs.

### Comparison with existing promoter predictors

DDM utilizes TSS prediction as a means to estimate NTLs. Many promoter predictors have been developed with the general aim to predict TSSs with certain levels of precision and positional accuracy [15]. However, none of these programs aims to identify NTLs. They are designed for different purposes [15], [16].

One could argue that the same goal that DDM is designed for can be achieved using existing promoter predictors. In principle, any promoter predictor that provides a very high sensitivity level (close to 100%) and predicts positionally accurate TSS locations can serve the purpose of estimating NTLs. To test if this is possible with the currently available promoter predictors, we evaluated several promoter prediction programs in their ability to accurately determine NTLs (i.e. how well they perform at or very near 100% sensitivity) and compared their performance to the NTL determination performance of DDM.

To make this comparison as fair as possible, we have created an independent test set that contains two subsets, HTSS_compare_ and RNDM_compare_. HTSS_compare_ contains 1000 randomly selected TSSs from the human TSS data. We also used 1000 random human DNA sequences, RNDM_compare_ (see Materials and Methods). We then retrained DDM with the remaining human TSS sequences and the remaining random DNA sequences. Consequently, the test set data (HTSS_compare_ and RNDM_compare_) was completely independent of the training set for DDM for this comparison analysis.

We evaluated programs from [15], [16], which we believe reflect a representative sample of current promoter predictors. We analyzed the performance of Promoter2.0 [17], NNPP2.2 [18], First Exon Finder [19], Eponine [20] and Fprom [21]. URLs for the online version of these tools can be found in the supporting information Document S1. N-SCAN [22] and McPromoter [23] do unfortunately only allow very limited online submission and thus were not included in our comparison experiment, while CpGProD [24], Dragon Promoter Finder [25], [26] and Dragon Gene Start Finder [27], [28] have specific design constraints that make them unsuitable for this comparison. The results of the comparison are summarized and discussed in the supporting information Document S1.

Based on the obtained comparison results, we observe that none of the promoter predictors described above achieves a performance that can match that of DDM regarding the ability to pinpoint NTLs. Details of the sensitivity and specificity levels achieved by the tested systems can be found in the supporting information Document S1.

### Application of DDM to estimate NTLs on showcase chromosomes

We applied the DDM algorithm to the forward and reverse strands of the human chromosomes 21, 22 and 4, which reflect an average, high and low GC-content with regard to the whole human genome. It was our aim to estimate what portion of the human genome can be regarded as certain NTLs and what portion of the genome would be estimated to contain 95% of all genuine TSSs. To estimate these two portions, we used two different threshold settings, 0.0 (produces ∼95% sensitivity on the training data) and −2.5 (produces 100% sensitivity on the training data). Firstly we observe that the number of NTLs is in correlation with the GC-richness of the chromosomes, as well as with the number of known genes [13] on these chromosomes. This means that the higher the GC-content of a chromosome or the reported gene density on a chromosome, the lower is the proportion of the chromosome estimated as NTLs and vice versa. However, one should note that in spite of this correlation, the NTLs estimated by our system are not confined to GC-poor regions and can also be found within GC-rich areas.

Chromosome 21 can be regarded as a showcase example, because it has approximately an average GC-content in comparison with the entire human genome. At threshold −2.5, 41.1% of the chromosome is demarcated as NTL. This allows us to estimate (based on currently available data) that roughly 40% of the human genome are completely or nearly completely devoid of TSSs. At threshold 0.0, we can demarcate 91.53% of human chromosome 21 as NTL. We showed that the remaining 8.47% of human chromosome 21 contains ∼92% of all genuine TSS we have in our dataset. Based on this we can hypothesize that roughly 10% of the human genome is home to 90% of all genuine TSSs. Also at threshold 0.0 we can annotate on human chromosome 22 (chromosome 4) 21.82% (4.13%) of the chromosome as TIAR and these TIAR regions contain 93.8% (83.6%) of all the genuine TSSs from our data on these chromosomes. The NTL and TIAR portions of the three chromosomes investigated at the two thresholds are shown in Table 2.

**Table 2:** NTL and TIAR of three showcase human chromosomes.

	Chromosome 21		Chromosome 22		Chromosome 4	
Threshold	NTL	TIAR	NTL	TIAR	NTL	TIAR
0.0	91.53%	8.47%	78.18%	21.82%	95.87%	4.13%
−2.5	41.1%	58.9%	27.2%	72.8%	46.84%	53.16%

### Example: DDM explains failed amplification by 5′-RACE

**Figure 3:**
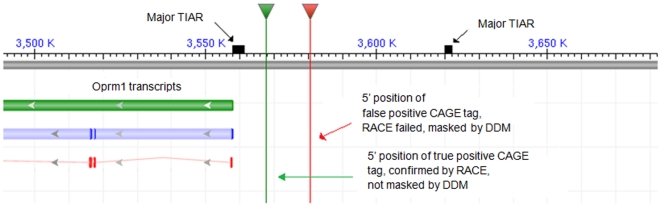
Application example. Illustration of DDM explaining failed amplification of 5′- RACE; true and false TSSs for mouse gene Oprm1 recognized by DDM.

To illustrate the usefulness of our tool for verifying whether transcripts derived by high-throughput experiments are 5′ complete, we considered the case of the CAGE tags between alternative TSSs in the gene Oprm1 in mouse (opioid receptor, mu 1; coordinates: chr10, negative strand 3308332..3557942; EntrezGene ID: 18390). DDM marks two major TIAR sections in the area. The larger one is about 3000 nt in size and contains the 5′ end of the gene (Figure 3). A smaller one is found about 60 Knt upstream of the gene and suggests the existence of an alternative TSS. DDM also marks numerous other positions as potential TSSs (not shown in Figure 3). A TSS at position 3557930 is supported by one CAGE tag (Fantom3 representative tag ID 122BA39P0901, undefined tissue library) and this TSS is within TIAR. This TSS was confirmed by 5′-RACE experiments in 4 out of 6 tissue samples supporting our prediction (primer T10F0065AF50) (for full details on 5′-RACE experiments see Online Supporting Materials for [5]). Contrary to this, a false positive TSS at position 3580940 indicated by one CAGE tag (Fantom3 representative tag ID 119BA53D1906, macrophage tissue library) could not be confirmed by 5′-RACE in any of the 6 tissues. RACE experiments with two different primers (T10F006553E1 and T10F006553F9) were conducted. Interestingly, this false-positive TSS is characterized as a NTL by DDM suggesting it is not likely to promote transcription. The NTLs surrounding these two CAGE tags are shown in the supporting information Document S1. Which positions are estimated to be NTLs and which positions are likely to initiate transcription in these surrounding areas is also indicated in the supporting information Document S1.

Because DDM operates with a resolution of a single nucleotide, NTLs and TIARs are sometimes small and clustered. For reasons of image resolution we therefore cannot show the complete NTLs and TIARs in Figure 3. However the exact demarcation around the CAGE tags from Figure 3 is shown in the supporting information Document S1.

Furthermore we have examined another 5 genomic positions where transcription is indicated by the existence of a single CAGE tag (Fantom3 representative tag IDs 120BA49K1606, 081AA66D1203, 069AE29I1002, 097AA30J2305 and 112BA90K2006). These CAGE tags are derived from adipose, liver, lung, macrophage and embryonic tissues, respectively. The existence of a transcript could not be confirmed by 5′-RACE for any of these positions. DDM places all but one of these positions within NTLs. The one which falls outside of an NTL region corresponds to the CAGE tag 112BA90K2006, which is marked as a potential TSS. We believe this is likely to indicate a false positive prediction by DDM, although it could be also a problem with the RACE experiment.

To illustrate NTLs and TIARs on a wider scale we have annotated the complete sequence of human chromosome 21 with DDM. The annotated sequence is shown in the supporting information Document S1.

## Discussion

We have developed a tool that enables us to very accurately determine the part of genomic locations that is highly unlikely to promote transcription initiation. While TSS prediction is a well-studied problem for which many tools exist, DDM constitutes the first tool that applies TSS prediction to estimate NTLs. We used this tool to make an initial annotation of NTLs in the human genome by processing three exemplary chromosomes. Our results suggest that over 40% of a mammalian genome consists of NTLs. The remaining portion of the genome (TIARs) should be understood to contain the vast majority of genuine TSS locations. It also contains those locations that were incorrectly labeled by our tool as potential TSSs.

In developing the algorithm we have exploited compositional properties of regions immediately surrounding genuine TSS locations. As we have determined our set of genuine TSS locations using at least two independent pieces of experimental evidence, our TSS sets for human and mouse can be considered very accurate. Moreover, since our TSS sets contain 113,814 human and 98,682 mouse TSS locations, these sets represent to the best of our knowledge the most comprehensive set of TSS locations derived in this way. The two sets contain many alternative TSSs for a large number of genes. In spite of the richness of our TSS data sets, we are aware that they cannot be considered ultimately complete.

Based on the large collections of transcription data available today, we have shown that transcription in mammals does not initiate randomly over the entire genome. Instead, only a small portion of the genome is likely to initiate transcription for a vast majority of transcripts. For *Homo sapiens* we estimate that no more than 10% of the genome is responsible for more than 90% of transcription initiation. Consequently, our results and tool can be used to demarcate in advance regions of greater interest for studies of transcription in mammals.

We hope that the DDM program is useful for researchers working on several types of problems, such as promoter identification, transcript and gene annotation, data curation from high-throughput experiments, wet-lab experiment designs and assessment of 5′ completeness of expressed sequences. All these aspects are of broader interest. Promoter identification, although considerably advanced [15], [29], still suffers from positional inaccuracy of prediction of actual TSS locations. The problem is circular to the accuracy of the dataset on which these systems are trained. This leads us to the second problem of a more accurate annotation of transcripts. Most frequently used methods for full-length cDNAs are Cap-trapper [30], [31] and Oligo capping [32]. Due to the specificity of sequences around mammalian TSSs (generally high GC% and strong secondary structures), under optimal conditions, over 90% of full-length cDNA can be generated with the rest of cDNAs being non-full-length [33]. Our system can help with the remaining problem of determining which cDNAs are in fact full-length and which ones are not. We see one of the main applications of DDM in the checking for 5′ completeness of transcripts generated in high-throughput experiments.

Bioinformatics approaches, microarray experiments, as well as other high-throughput data are prone to false-positives. The genuine TSS locations have to be confirmed through wet lab experiments (Northern hybridization, RACE, RT- or quantitative PCR) and possibly by multiple pieces of evidence. Most of low-throughput but high-confidence experimental techniques require a priori knowledge of specific genomic regions for probe or oligonucleotide primer design. The design of more accurate probes, by applying DDM before experimental validation, would thus be of advantage. The examples on 5′ RACE that we provided above illustrate this point. The novelty in using DDM for this purpose is that a NTL characterization by DDM is highly reliable, because at or very near 100% TSS prediction sensitivity the system produces no or very few false-negatives, while at the same time the false-positive rate of DDM is superior to other tested promoter predictors at this level of sensitivity.

### Conclusions

We presented a new method for estimating locations that are unlikely to initiate transcription in mammalian genomes. This method applies TSS prediction at very high sensitivity levels. The algorithm's ability to demarcate a significant portion of the genome as containing only a minimal fraction of genuine TSS locations while retaining the vast majority of genuine TSSs in the remaining regions, allows the focusing of research attention to narrow segments of the genome that could otherwise be difficult to identify. The great advantage of our algorithm is that it can identify locations not likely to initiate transcription at the resolution of a single nucleotide. The server with our algorithm is freely accessible at http://cbrc.kaust.edu.sa/ddm/.

## Materials and Methods

### Transcription Start Sites

Two highly accurate TSS sets for mouse (genome built mm8) and human (genome built hg18) and their respective surrounding sequences covering [−100,+100] relative to these TSSs were compiled. If the first 5′ nucleotide of the CAGE tag (FANTOM3) exactly coincided with the first 5′ nucleotide of at least one flcDNA (UCSC and Fantom3), or at least one mRNA (UCSC), the TSS determined by this tag is selected as ‘genuine’. Thus, all TSS locations selected in this way are supported by at least two independent pieces of evidence. No minimum distance between neighboring TSSs was enforced as long as two pieces of evidence were present at a location with no mismatch allowed. Webelieve that the resulting sets of TSSs have an extremely high accuracy. Sequences that contained ambiguous characters (‘N’) were excluded. In this way we compiled a mouse reference TSS set containing 98,682 sequences (MTSS) and a human reference TSS set containing 113,814 sequences (HTSS). We have split the HTSS set into two disjoint parts, HTSS_tc_ and HTSS_compare_. HTSS_compare_ is obtained by randomly selecting from the HTSS set a subset of 1,000 TSS locations. For these TSS locations we have extracted the sequences covering [−800, +800] relative to the TSS location. HTSS_compare_ is used for comparison of different promoter predictors.

It has been established by [34] and [35] that within promoter regions there exist many alternative TSSs that are often located within a few nucleotides from each other. Here we regard these TSS as separate, even if they are residing on neighboring nucleotides. Although TSSs that are located very close to each other are likely to transcribe the same transcriptional unit, even the most minimal difference in the location of the TSS leads to the production of a slightly different transcript. Since we are aiming to pinpoint the exact location of TSS and not only the approximate location of a core promoter, we therefore regard these transcription events as separate. Furthermore even a small positional difference between two TSSs causes the surrounding area of the TSS to be different, with features of this area residing at different locations with regard to the TSS. While this approach has the effect that NTLs and TIARs in some cases appear in a clustered fashion on the chromosomal sequence, we believe that this could reflect the actual biological situation with regard to transcription initiation.

### Other sequences

A set of randomly selected DNA sequences from human and mouse was compiled for the reference ‘negative’ set. These DNA sequences were of 200 nt length selected randomly from all human and mouse chromosomes with the number of sequences proportional to the length of the chromosomes. Sequences that contained ambiguous characters (‘N’) were discarded. If the 5′ end of a CAGE tags fell within [−10, +10] relative to the center of the sequences, the sequence was also discarded. In total we selected 110,000 random human DNA sequences (RNDM) and 100,000 random mouse DNA sequences. In the same manner we extracted additional 1,000 human DNA sequences of length 1600 nt to be used as a negative set for comparison with promoter predictors. This set was called RNDM_compare_. This means that RNDM_compare_ and the negative set (RNDM) used for training of DDM are disjoint.

### Algorithm

To achieve the highest possible accuracy, the presented algorithm utilizes a four-stage daisy-chained filtering method. Sequences of length 200 are examined and have to be classified by all four stages as a potential TSS in order not to be NTL. Using a different filtering method at each stage we are able to exploit different compositional features of the sequence under examination. Thus, the overall method achieves the very high discrimination between locations likely and locations not likely to initiate transcription.

All steps are performed either on the entire available data sets or on the training part of the data during the 4-fold CV.

#### Boundaries of k-mer distribution and frequencies of k-mers

**Figure 4:**
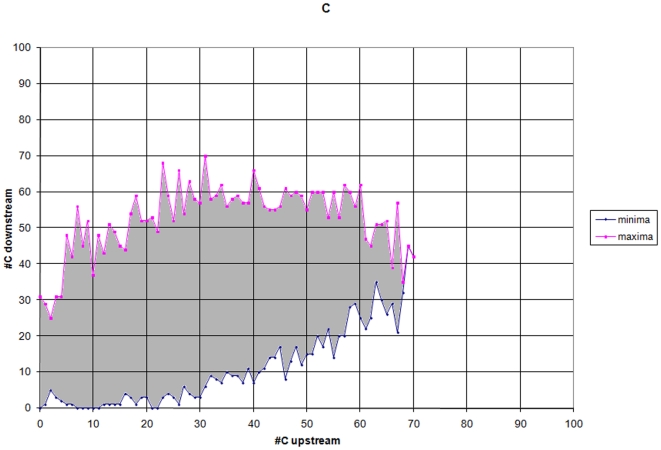
Constraining sequence properties (nt C). Constraining boundaries for occurrences of 1-mer ‘C’.

We considered a total number of 1,364 k-mers of length 1–5 (Table 3). We determined the number of occurrences u of each k-mer K in the upstream segment [−100, −1], as well the number of occurrences d of K in the downstream segment [+1,+100] and recorded these two numbers. Both values u and d are from the interval [0, 100+1-k] where k denotes the length of k-mer K. For every sequence in MTSS and HTSS with an upstream occurrence u of k-mer K we determined the minimum, min(d), and maximum, max(d), occurrence of K downstream of TSS. For every sequence in MTSS and HTSS with an downstream occurrence d of k-mer K we determined the minimum, min(u), and maximum, max(u), occurrence of K upstream of TSS. For every k-mer K, the collection of all points defined by (min(d),u) and (max(d),u), as well as (d,min(u)) and (d,max(u)), define boundaries of the region that contains all TSS locations. (Example Figure 4, region of all TSS shown in grey). A particular TSS characterized by (u1,d1) for k-mer K will be recognized, if for u1 we get min(d) ≤d1≤ max(d), and for d1 we obtain min(u) ≤u1≤ max(u). A sequence is considered to contain a TSS on position +1 if for all 1,364 k-mers it satisfies all above constraint conditions.

**Table 3:** Statistics on k-mers used in development of the algorithm.

k-mer length	Number of k-mers	Cumulative number of features used
1	4	4
2	16	20
3	64	84
4	256	340
5	1024	1364

#### [−10,+10] PWM thresholding

The sets MTSS and HTSS are divided into 16 subsets characterized by different dinucleotides at positions [−1,+1]. For each of these subsets, we extracted all sequences of length 20 nt covering the region [−10,+10], and for each of the 16 such subsets we constructed a position weight matrix (PWM) following [36]. The PWM of a given subset is subsequently used to determine the PWM scores s_s_ of all [−10,+10] sequences in the subset. Out of these scores the minimum score s_min_ is selected. A sample is considered to contain a TSS on position +1 if its associated PWM score s_s_≥s_min_ in the respective subset.

#### LDF 40

MTSS and HTSS are divided into 16 subsets, as described above. The complete TSS region [−100,+100] for all TSSs in a given subset is divided in 40 consecutive non-overlapping sections of length 5 nt. For each of these 40 sections we determined a PWM as previously described, using all sequences from a given subset of HTSS and MTSS respectively. For each of the sequences from all 16 subsets of HTSS and MTSS we determine a feature vector comprising of 40 PWM scores. Each score was determined using all 40 sections of the sequence and the corresponding PWM. This way we produced 16 sets of ‘positive’ data with one 40-element feature vector for each sample. We processed the ‘negative’ data with the same PWMs derived from the MTSS and HTSS subsets to create 16 sets of ‘negative’ data with one 40-element feature vector for each sample.

Linear discriminant analysis [37] is used on these sets of ‘positive’ and ‘negative’ data to determine 16 linear discriminant functions (LDFs), one for each of the 16 subsets. An LDF value is calculated using 40 coefficients c_i_, i = 1,2, …,40, plus one constant c_0_. All sequences in HTSS and MTSS are subjected to the LDF that corresponds to the dinucleotide at positions [−1;+1] and the score s_LDF_ is calculated for each sequence (s_LDF_ = c_1_x_1_+…+c_40_x_40_+c_const_, where x_i_ are the corresponding scores of the respective PWMs). A threshold value is determined for each of the 16 subsets in MTSS and HTSS by selecting LDF_min_ so as to preserve 100% sensitivity in the recognition of real TSSs.

A sample is considered to contain a TSS on position +1 if LDF_sample_ ≥ LDF_min_ for the respective subset. Otherwise, the sample is classified as not containing a TSS on +1.

#### SVM

The sets MTSS and HTSS and the ‘negative sets’ are processed as described above to produce positive and negative data containing 40 values for each sample. A support vector machine (SVM light: http://svmlight.joachims.org/) with a radial basis kernel function is trained as a classifier. The radial basis gamma value 1.28 delivered the highest accuracy for our data. The class for sequences containing a genuine TSS is labeled 1, the class for random non-TSS DNA sequences is labeled −1. The two resulting models M_HS_ and M_MM_ are derived.

A threshold value t_SVM_ is then applied. A sample is considered to contain a TSS at position +1 if the SVM score s_SVM_>t_SVM_.

The threshold t_SVM_ is the only adjustable input parameter to the tool implemented on our web server. It can be used to manipulate the sensitivity/specificity behavior of the algorithm (See Results and Discussion section for details). All other parameters of the algorithm are fixed at a level that experimentally provided maximum sensitivity. In particular, the threshold values for [+10, −10] PWM and for LDF40 functions were fixed at levels that allow us to retain 100% of true TSSs. Although it is possible to use these thresholds to manipulate the sensitivity/specificity behavior of the algorithm, we have experimentally determined the SVM to be the step that allows the most beneficial trade-offs, and moreover, contribute to the simplicity of the parameter adjustment process. Because the SVM classification of sequences is also computationally the most time consuming, it is beneficial for the overall speed of the algorithm to place it at the end of the daisy chain.

## Supporting Information

Document S1 Supplementary materials for manuscript.
(0.08 MB DOC)
