# Supplementary material for: High Sensitivity TSS Prediction: Estimates of Locations Where TSS Cannot Occur
Source: PLoS One. 2010 Nov 15;5(11):e13934. doi: 10.1371/journal.pone.0013934 (PMC2981523; doi:10.1371/journal.pone.0013934)
Supplement: Document S1 — Supplementary materials for manuscript. (0.08 MB DOC) [file pone.0013934.s001.doc]

## SUPPLEMENTARY MATERIAL

## for

## “High sensitivity TSS prediction: Estimates of locations where TSS cannot occur”

by

Ulf Schaefer, Rimantas Kodzius, Chikatoshi Kai, Jun Kawai, Piero Carninci, Yoshihide Hayashizaki and Vladimir B Bajic

**Comparison of performance of DDM with existing promoter prediction tools**

A set of 1000 sequences from human covering [-800, +800] relative to a known true TSS (HTSS_compare_) and a set of randomly chosen human sequences of length 1600 nt RNDM_compare_ were used in the comparison.

We have specifically retrained DDM with TSS data HTSS_tc_ which does not contain HTSS_compare_, and with random DNA data RNDM that does not contain RNDM_compare_. The obtained DDM model was used in the comparison. Neither data from RNDM_compare_ nor data from HTSS_compare_ was therefore known to DDM system during the training process.

We conducted two tests. For the first we allowed a mismatch of ±100 nucleotides for a prediction to be counted as correct (test A). For the second we counted only those predictions as correct that predict the known TSS with no mismatch (test B). A negative prediction was regarded as correct if there was no prediction within 100 nucleotides of position 801 for each sequence in RNDM_compare_ (test A) or if there was no prediction at 801 exactly (test B). The results of our experiments are described below and summarized in table S2. The test B is thus far more stringent than test A. We highlight that our DDM tool is operating under conditions as used in test B.

On HTSS_compare_ and RNDM_compare_ DDM achieves a sensitivity of 99.8% and a specificity of 40.1% at threshold -2.0 with no mismatch in the predicted location of TSSs. For comparison, in Table S3 we show the performance of DDM with resulting sensitivity and specificity levels when no mismatch in predicted TSS locations is required.

**Promoter2.0**

Promoter 2.0 does not allow the setting/tuning of any threshold. For test A we achieved a sensitivity of 22.5% and a specificity of 86.6% on HTSS_compare_ and RNDM_compare_ respectively. Promoter2.0 does not provide predictions with one nucleotide resolution, so test B cannot be applied for this tool.

**NNPP2.2**

For test A we determined the threshold for which we achieve 100% sensitivity on HTSS_compare_ for NNPP2.2 to be t=0.12. For this value of t we observe a specificity of 4% on RNDM_compare_ in test A. For test B we observe a sensitivity of 21% and a specificity of 95% for the same value t=0.12.

**First Exon Finder**

We used the lowest available threshold t=0.2 for all probabilities. For test A we achieved a sensitivity of 40.7% and a specificity of 98.6% on HTSS_compare_ and RNDM_compare_ respectively. First Exon Finder does not provide predictions with one nucleotide resolution, so test B cannot be applied for this tool.

**Eponine**

We used the lowest available threshold t=0.9. For test A we achieved a sensitivity of 34.3% and a specificity of 91.4% on HTSS_compare_ and RNDM_compare_ respectively. Eponine does not provide predictions with one nucleotide resolution, so test B cannot be applied for this tool.

**Fprom**

We used the thresholds for which the authors report a sensitivity of 100.0% for non-TATA-box promoters and TATA-box promoters respectively (-9.496 and -6.766). For test A we achieved a sensitivity of 59.3% and a specificity of 99.4% on HTSS_compare_ and RNDM_compare_ respectively. For test B we observed a sensitivity of 2.4% and a specificity of 100.0%

**N-SCAN**

N-SCAN does not allow the submission of multiple sequences simultaneously and due to size of our test data we could not conduct any tests.

**McPromoter**

McPromter does not allow the submission of multiple sequences simultaneously and due to size of our data we could not conduct any tests. The author of this tool does however report that McPromoter has a sensitivity of 65% at the highest available sensitivity level.

**Discussion of performance comparison**

In the attempt to show that the available promoter predictors are not suitable to predict NTL with an acceptable level of accuracy we compared our DDM system with several such tools. Since it is only possible to meaningfully speak of NTL and TIAR in terms of a TSS classification that was conducted with sensitivity of or very near 100% we attempted to simulate this situation with the existing promoter predictors. Our goal is not to claim how good DDM is for the task of predicting TSSs, but how well it is performing in assessing NTL. At the same time we claim that current promoter predictors in the versions available to the general public, are just not useful for this task. Three of the promoter predictors examined in [1,2] are by design unsuitable for a comparison with DDM. These tools are CpGProD [3], Dragon Promoter Finder [4,5] and Dragon Gene Start Finder [6,7]. CpgProD is restricted to prediction of CpG-island related promoters, while Dragon Promoter Finder and Dragon Gene Start Finder determine TSS based on averaging of the strong predictions, thus generating a TSS prediction that is located close to the real TSS but unlikely to represent the real TSS. Other systems have other types of restrictions.

The general comparison setup was based on the use of a test set that is completely independent of the training set used to derive the DDM model for the comparison test. Furthermore, to be able to estimate NTL, the promoter predictors should be able to operate at sensitivities of ~100%. Not all promoter predictors we had access to have the possibility to adjust their tunable parameters to achieve this level of sensitivity, and thus we claim that they are not really suitable for the task of estimating NTL. However, for those promoter predictors that allow adjustment of parameters to make them operate at high sensitivity levels we made such adjustments. It is important to note that after this intervention, such promoter predictors have reached an ‘extreme setting’ relative to their typical mode of operation.

The observed differences in performance comparison results stem from several factors. First, DDM is capable of separating NTL and TIAR predictions at the level of a single nucleotide, because the DDM algorithm is trained to pinpoint the actual TSS location. Promoter predictors frequently only indicate a region in which they expect a TSS to be present, thus reducing the resolution of the prediction dramatically. Only two of the promoter predictors we tested, NNPP2.2 and Fprom, are capable of pinpointing the TSS location exactly. All other predictors we used give only an interval in which they claim a TSS location is. The comparison with DDM (test B) when the exact TSS location is to be predicted, shows that both NNPP2.2 and Fprom are not recognizing a significant portion of the real TSS locations, making them unsuitable for NTL estimation. Another issue is that the design goals of promoter predictors are likely to be different from the design goal of DDM. Promoter predictors, on the other hand, normally aim at maximized balanced sensitivity and specificity, usually sacrificing sensitivity in lieu of specificity. Systems tuned in such a way are not necessarily suitable for determining NTL, as one needs to be certain that all (or the vast majority of) TSSs are correctly recognized. It is for this reason that the settings of the promoter predictors had to be adjusted in order for them to operate with the highest possible sensitivity. We highlight that the comparison results have to be interpreted with these issues in mind.

**Table S1: URLs of promoter prediction tools.**

| Tool | URL |
| --- | --- |
| Promoter2.0 | http://www.cbs.dtu.dk/services/Promoter/ |
| NNPP2.2 | http://www.fruitfly.org/seq_tools/promoter.html |
| First Exon Finder | http://rulai.cshl.org/tools/FirstEF/ |
| Eponine | http://servlet.sanger.ac.uk:8080/eponine/ |
| Fprom | http://www.softberry.ru/berry.phtml?topic=fprom  &group=programs&subgroup=promoter |

Table S2: Performances of Promoter prediction tools

|  | Test A (±100 nt mismatch) | | | Test B (no mismatch) | |
| --- | --- | --- | --- | --- | --- |
| Tool | SE | SP | threshold | SE | SP |
| Promoter2.0 | 22.5% | 86.6% | n/a | n/a | n/a |
| NNPP2.2 | 100.0% | 4.0% | 0.12 | 21.0% | 95.0% |
| First Exon Finder | 40.7% | 98.6% | 0.2 | n/a | n/a |
| Eponine | 34.3% | 91.40% | 0.9 | n/a | n/a |
| Fprom | 59.3% | 99.4% | 0.0 | 2.4% | 100.0% |

Table S3: Test B: performance of DDM on HTSS_compare_ and RNDM_compare_ with no mismatch

| Sensitivity | Specificity |
| --- | --- |
| 2.40% | 100.00% |
| 4.0% | 99.90% |
| 21.00% | 99.50% |
| 22.50% | 99.20% |
| 34.30% | 99.10% |
| 38.40% | 98.60% |
| 40.70% | 98.50% |
| 53.3% | 98.2% |
| 85.50% | 95.00% |
| 91.20% | 91.40% |
| 92.30% | 86.60% |
| 95.20% | 58.70% |
| 99.80% | 4.00% |
| 100.00% | n/a |

**DDM estimates of NTL around CAGE tags T10F0065AF50 and T10F006553E1**

CAGE tags are shown in bold and yellow highlight, masked positions are shown in lower case letters and strikethrough, potential TSS are shown in capital letters

>122BA39P0901 mm5 chr10 – 3559800.. 3559350

~~aaacaacctaatgcatggttgttacaaagaaac~~ttag~~ag~~tctctc~~t~~tccc

accctctctgcagcatccccgcttctgctccccccccccctaccccaatt

tacactcctttacacggaagtgcatagagactgaggaggctgattctgag

ttgctttgcgcacaatccactccttctctctcctccctcccctctagcct

ctggatccctcacagcccatgctccctcccttccactc**AGAGAGTGGCGC**

**TTTGGGG**atgctaaggatgcgcctccgtgtacttctaaggtgggaggggg

ctacaagcagaggagaatatcggacgctcagacgttccattctgcctgcc

gctcttctctggttccactagggcttgtccttgtaagaaactgacggagc

ctagggcagctgtgagaggaagaggctggggcgcctggaacccgaacact

>119BA53D1906 mm5 chr10 – 3583000.. 3582500

~~tgaaatgtaagtaaagaaattatctaataaaaatatataaaataaataaa~~

~~taaatggactcatgtgtgtgtgtgtgtgtgagagagagagagagagagag~~

~~agagagcgatctgttgatagtttggtgttttgttttctttgggggtatgg~~

~~tttgttttgtttccttggtttttggggggtatcgctgtattgggttgttt~~

~~ggtttttcaggaaacttaaagttg~~**~~ggtgggtagggaccaggg~~**~~aaggattt~~

~~tgaagaacatggagaatggggagaatatggtcaacatatatttaaattta~~

~~aaatagttttaaataataaaaatataatataggtaatataatcattaaaa~~

~~atctgccataaataaggagttaaaggaaattacaaagtaagttaaaaaaa~~

~~caaaaagcaatgtcatccatatagatacatatatatataactaaatataa~~

~~tatatagcataaaagaaagtcaacttcacaaaagtaaatatgataattaa~~

**Whole chromosome NTL and TIAR regions at 3 levels of Sensitivity**

Human chromosome 21 (forward strand, genome version hg18) is available for download from <http://cbrc.kaust.edu.sa/ddm/download/>. We present the chromosomal sequence with NTL regions masked (character ‘-‘) at the sensitivity levels of 95%, 99% and 100%.

**References**

1. Bajic VB, Tan SL, Suzuki Y, Sugano S (2004) Promoter prediction analysis on the whole human genome. Nature biotechnology 22: 1467-73. doi:10.1038/nbt1032

2. Bajic VB, Brent MR, Brown RH, Frankish A, Harrow J, et al. (2006) Performance assessment of promoter predictions on ENCODE regions in the EGASP experiment. Genome biology 7 Suppl 1: S3.1-13. doi:10.1186/gb-2006-7-s1-s3

3. Ponger L, Mouchiroud D (2002) CpGProD: identifying CpG islands associated with transcription start sites in large genomic mammalian sequences. Bioinformatics (Oxford, England) 18: 631-3.

4. Bajic VB, Seah SH, Chong A, Zhang G, Koh JL, et al. (2002) Dragon Promoter Finder: recognition of vertebrate RNA polymerase II promoters. Bioinformatics (Oxford, England) 18: 198-9.

5. Bajic VB, Chong A, Seah SH, Brusic V (2002) An Intelligent System for Vertebrate Promoter Recognition. IEEE Intelligent Systems. 64-70

6. Bajic VB, Seah SH (2003) Dragon gene start finder: an advanced system for finding approximate locations of the start of gene transcriptional units. Genome research 13: 1923-9. doi:10.1101/gr.869803

7. Bajic VB, Seah SH (2003) Dragon Gene Start Finder identifies approximate locations of the 5' ends of genes. Nucleic acids research 31: 3560-3.
